# Food Addiction and Tobacco Use Disorder: Common Liability and Shared Mechanisms

**DOI:** 10.3390/nu12123834

**Published:** 2020-12-15

**Authors:** Laurie Zawertailo, Sophia Attwells, Wayne K. deRuiter, Thao Lan Le, Danielle Dawson, Peter Selby

**Affiliations:** 1 Department of Pharmacology and Toxicology, University of Toronto, 1 King’s College Circle, Toronto, ON M5S 1A8, Canada; 2 Centre for Addiction and Mental Health, 1025 Queen Street West, Toronto, ON M6J 1H4, Canada; sophia.attwells@camh.ca (S.A.); wayne.deruiter@camh.ca (W.K.d.); thaolan.le@gmail.com (T.L.L.); danielle.dawson@camh.ca (D.D.); peter.selby@camh.ca (P.S.); 3 Department of Family and Community Medicine, University of Toronto, 500 University Ave., Toronto, ON M5G 1V7, Canada; 4 Department of Psychiatry, University of Toronto, 250 College St., Toronto, ON M5T 1L8, Canada; 5 Dalla Lana School of Public Health, University of Toronto, 155 College St., Toronto, ON M5T 3M7, Canada

**Keywords:** food addiction, nicotine, tobacco use disorder, comorbidity

## Abstract

As food addiction is being more commonly recognized within the scientific community, parallels can be drawn between it and other addictive substance use disorders, including tobacco use disorder. Given that both unhealthy diets and smoking are leading risk factors for disability and death, a greater understanding of how food addiction and tobacco use disorder overlap with one another is necessary. This narrative review aimed to highlight literature that investigated prevalence, biology, psychology, and treatment options of food addiction and tobacco use disorder. Published studies up to August 2020 and written in English were included. Using a biopsychosocial lens, each disorder was assessed together and separately, as there is emerging evidence that the two disorders can develop concurrently or sequentially within individuals. Commonalities include but are not limited to the dopaminergic neurocircuitry, gut microbiota, childhood adversity, and attachment insecurity. In addition, the authors conducted a feasibility study with the purpose of examining the association between food addiction symptoms and tobacco use disorder among individuals seeking tobacco use disorder treatment. To inform future treatment approaches, more research is necessary to identify and understand the overlap between the two disorders.

## 1. Introduction

Smoking and obesity are the two most prevalent causes of preventable chronic disease morbidity and mortality worldwide [1,2]. Smoking and food addiction are both maladaptive behaviors in which an individual experiences loss of control and compulsive engagement in the behavior despite known harmful consequences. We have reached a pivotal time for understanding food addiction, similar to a time when tobacco use disorder was perceived as habit forming and not addictive. While food addiction does meet several of the American Psychiatric Association’s diagnostic criteria outlining substance use disorder [3], more research is necessary to determine if certain foods are addictive and how to prevent and treat this condition.

The concept of food addiction represents a relatively new domain of research [4]. Food addiction refers to an “eating behavior involving the overconsumption of specific foods in an addiction-like manner”. While the term “food addiction” was introduced to scientific literature in 1956 [5], research investigating the mechanisms, neurobiology, and genetics of food addiction was not pursued until the early 2000s. Food addiction in relation to certain foods has not been widely accepted or studied. As such, the diagnostic criteria of food addiction are not well established and are not formally recognized by the American Psychiatric Association as either a substance use disorder or a behavioral disorder in the Diagnosis and Statistical Manual of Mental Disorders 5 (DSM 5) [3]. Regardless, the criteria for food addiction have been modeled from substance use disorder criteria outlined in the DSM 5 [6].

In the DSM 5, substance use disorder is defined as a complex condition manifested by compulsive substance use despite harmful consequences. Furthermore, regular substance use may develop dependence and tolerance. The DSM 5 currently lists nine distinct disorders (e.g., tobacco use disorder and alcohol use disorder), but nearly all substances are diagnosed on the basis of the same overarching criteria [3]. Hallmark symptoms of food addiction include loss of control and frequent overconsumption, desire or repeated failed attempts to reduce or stop consumption, increased time spent in activities necessary to obtain and eat food, giving up on important activities such as physical exercise, continued consumption despite physical or psychological problems, and clinically significant impairment or distress [7].

Currently, limited research exists pertaining to food addiction and how it relates to other substance use disorders. Given that both unhealthy diets and smoking are among the leading risk factors for all-cause disability-adjusted life years, total deaths, and years lived with disability [8], a greater understanding of how food addiction and tobacco use disorder overlap with one another is necessary [9]. Furthermore, the role of food addiction in the common but understudied phenomenon of post-cessation weight gain among smokers trying to quit has not been researched. The purpose of this narrative review is to present and summarize the most up-to-date research findings on the prevalence, biology, psychology, and treatment of tobacco use disorder and food addiction as individual disorders, identifying overlap and commonalities that may help inform treatment approaches. It is important to understand each individual disorder in the context of the other since there is emerging evidence that the two disorders can develop concurrently or sequentially within individuals. To provide background on food addiction and tobacco use disorder, this review first discusses the prevalence of these disorders, measurements that assess severity, and theories of food addiction. This review then covers various subtopics such as biology, gut microbiome, psychosocial factors, and treatment options. The studies examined for this review mainly focus on food addiction rather than obesity and eating disorders, as these are two separate and complex conditions.

## 2. Methods

To locate relevant publications on the relationship between food addiction and tobacco use disorder, the following databases were searched: Ovid Medline, PsycInfo, and Embase. The Medline search strategy included both relevant medical subject headings (MESH) and keywords for the concepts of food addiction and tobacco use disorder/cessation. Food addiction-related terms included “food adj3 addict”, “eat adj3 addict”, “food adj3 dependence”, “food use disorder”, “compulsive eating”, “Yale Food Addiction Scale”, “obes”, “diabetes”, and “binge eating disorder”. Smoking terms included “tobacco use disorder”, “tobacco depend”, “nicotine”, “((nicotine or tobacco or smoking) adj3 (cessation or quit or quitting or quits or give up or giving up or stop or stopping or stopped or stops))”, “((nicotine or tobacco or smoking) adj3 withdraw)”, and “smoking cessation/or tobacco use cessation”. The Medline search strategy was adapted for the controlled vocabulary of the other databases searched. All of the search results were limited to English language publications, but no date or study type limitations were applied. Furthermore, the database search was complemented by a manual review of reference lists from the retrieved articles. Articles related to the purpose of this narrative were retrieved from the database search. Studies examining eating disorders and obesity in the absence of food addiction were not reviewed as this was outside of the scope of this review.

## 3. Discussion

### 3.1. Prevalence

#### 3.1.1. Prevalence and Severity Measurement of Tobacco Use Disorder

In 2017, 15% of the Canadian population (about 4.6 million people) were current smokers. Within that population, 11% and 4% reported being daily smokers and occasional smokers, respectively [10]. Daily smokers averaged approximately 14 cigarettes per day, with a higher percentage of smokers being male (17%) than female (13%) [10]. Furthermore, in 2018, nearly 34% of Canadian youth reported trying an electronic cigarette (e-cigarette) at least once in their lifetime [11]. Evidence suggests that e-cigarette use in youth increases the risk of developing tobacco use disorder later in life [12]. Similar statistics are reported in the United States, where approximately 12% of the population (or 40 million people) are daily smokers [13].

The DSM 5 replaced the DSM-IV’s categories of nicotine abuse and dependence with tobacco use disorder. Within this context, problematic patterns of tobacco use must cause significant impairment and distress for at least two symptoms within a 1 year period to be considered a disorder. Other common measures of nicotine dependence severity include the Fagerstrom Test for Nicotine Dependence (FTND), Heaviness of Smoking Index (HSI), and time to first cigarette (TTFC). The FTND contains six questions scored 0–3 for a total possible score of 0–10. Higher scores indicate greater the physical dependence on nicotine. Similarly, HSI contains two measures: TTFC of the day and daily consumption of cigarettes. These metrics have been used to predict behavioral and biochemical indices of smoking, including ability to quit and cancer incidence and mortality [14]. Both measures are reliable over time and are important predictors of quitting [15,16].

#### 3.1.2. Prevalence and Severity Measurement of Food Addiction

Food addiction is commonly measured using the Yale Food Addiction Scale (YFAS) [17], a 25-item tool developed in accordance with the substance dependence criteria of the DSM-IV [7,18]. The YFAS applies these criteria to the concept of food addiction for the purpose of identifying individuals who possess a predisposition toward the overconsumption of highly palatable foods within the previous 12 months [7,19]. The YFAS is capable of providing two scoring measures: (i) a cutoff score (yes/no) is achieved when an individual fulfills a minimum of three criteria and satisfies a clinical significant impairment or distress criterion, and (ii) a symptom count (0–7) in which the total number of endorsed criteria, with the exception of clinically significant impairment or distress criteria, is added together [7,18]. The YFAS represents a valid and reliable tool in identifying individuals who meet criteria for food addiction [7,20]; however, a major limitation of the YFAS is the reliance on self-reporting of symptoms. To date, there are no biological measurements to confirm the presence or absence of food addiction. Therefore, in conjunction with the lack of formal recognition by the DSM 5, a clinical diagnosis for food addiction does not exist at this time.

With the release of the DSM 5, criteria for diagnosing substance-related and addiction disorders have changed. Consequently, the 35-item YFAS 2.0 was developed to accurately align the concept of food addiction with the new DSM 5 substance use disorder criteria [21]. Another adaptation, the modified version of the YFAS (mYFAS), is an abbreviated nine-item version of the YFAS. Within the mYFAS, each of the seven DSM-4 substance dependence criteria is represented by one question [18]. The remaining two items of the mYFAS pertain to whether food or eating causes an individual clinically significant impairment or distress [18]. Both YFAS 2.0 and mYFAS have demonstrated marginal to good psychometric properties [20,22].

The observed prevalence rate of food addiction in a meta-analysis of 20 studies from North American and European countries was 19.9% (range: 16.3% to 24.0%) [23]. In a more recent meta-analysis of 36 articles, Burrows et al. (2018) concluded that the prevalence of individuals with mental health symptoms who met the cutoff for food addiction was 16.2% (range: 13.6% to 19.3%) [22]. A sex difference was also observed, where higher prevalence of food addiction appeared among females (12.2%) compared to males (6.4%) [23].

Although many individuals may not meet the threshold for food addiction, it is not uncommon for individuals to report specific symptoms. On average, individuals with mental health symptoms endorse approximately three symptoms for food addiction [22,23]. Of the seven YFAS symptoms for food addiction, “persistent desire or unsuccessful attempts to cut down or control eating” is one of the most frequently endorsed symptoms of food addiction [19,23,24].

#### 3.1.3. Theories of Food Addiction

Several theories link specific high-caloric and palatable foods to food addiction. Similar to addictive substances, these foods exist on a spectrum of addiction [7]. For example, individuals meeting the cutoff for food addiction convey significant problems with highly palatable foods such as chocolate, doughnuts, cookies, cake, candy, white bread, pasta, rice, crackers, French fries, and hamburgers compared to their counterparts without food addiction [25,26]. In addition, more frequent consumption of hamburgers, candy bar, milk chocolate, butter, pizza, and low-calorie beverages was associated with meeting the cutoff for food addiction [26]. Conversely, more frequent consumption of dark chocolate, homemade cookies, white rice, and sugar-sweetened beverages was negatively associated with food addiction [26]. Other theories suggest that food itself is not addictive, but the manner in which the food is consumed is an addictive behavior. Specifically, the repetitive behavior of food restriction and dieting leads to periods of overeating and binging. While controversy surrounds the addictive properties of foods, it is important to consider that tobacco took several decades before it was declared an addictive substance [27]. Addictive substances including alcohol, tobacco, and cannabis have gained acceptance at some point and it was not until these substances were recognized as being addictive that society implemented changes that would provide opportunities for individuals to receive treatment [27].

### 3.2. Biology

#### 3.2.1. Neurobiological Parallels between Food Addiction and Tobacco Use Disorder

Within the past several decades, neuroimaging has allowed the quantification of specific proteins and neurotransmitter receptors, the investigation of food and tobacco cues and their effects on neural activation, and the investigation of the integrity of gray and white matter, using positron emission tomography (PET), functional magnetic resonance imaging (fMRI), and structural MRI, respectively. Although no specific neuroimaging study has investigated the neural correlates of tobacco use disorder with comorbid food addiction, when investigated separately, the effects of food addiction on the brain often resemble those of tobacco use disorder. Specifically, highly palatable foods, such as those with high fat and sugar content, can activate the dopaminergic (DAergic) reward pathways [7,28,29,30], and specific conditioned food cues such as its sight, smell, and taste may trigger the desire or craving of eating [31]. This suggests that common neural substrates exist for both food and tobacco use disorder, both of which depend on DAergic pathways.

To date, no PET studies have investigated dopamine (DA) receptor availability in individuals with food addiction. Although obesity and food addiction are distinct disorders, most neurobiological literature surrounding food addiction is derived from obesity studies since the two conditions often co-occur. The first human neuroimaging study to examine striatal DA D2 receptor availability in relation to obesity was Wang et al. 2001 [32]. Measured by [^11^C]raclopride PET, striatal DA D2 receptor availability was significantly lower in obese individuals compared to healthy controls, and body mass index (BMI) was negatively correlated with D2 receptor availability. Similarly, low levels of DA are often reported in individuals addicted to drugs including cocaine [33], alcohol [34], opiates [35], and nicotine [36], and low DA receptor levels are associated with addictive behaviors irrespective of food or addictive drugs [32]. DA deficiency may perpetuate pathological eating to replenish the mesolimbic DAergic pathway, and feeding has been shown to increase extracellular DA levels in the nucleus accumbens (NAcc) [37], a region thought to contribute to the reinforcing effects of euphoria [38]. It is possible that chronic overconsumption of food leads to increases in DA, resulting in DA D2 receptor downregulation. This produces a feed-forward cyclical pattern where overconsumption of food must then be sustained to replenish DA levels to avoid food cravings and withdrawal symptoms [39]. 

DA receptor availability in individuals with tobacco use disorder has been extensively investigated with [^11^C]raclopride PET. For example, a 26% to 37% reduction in binding potential, indicative of greater DA release, was observed in the left ventral caudate, NAcc, and left ventral putamen in cigarette smokers compared to nonsmokers [40]. In contrast, several other PET studies using tobacco cigarettes and alternative methods of nicotine administration, such as nicotine nasal sprays and nicotine gum, found no significant changes in binding potential within smokers [41,42,43,44]. More recent nonhuman primate PET studies found [^11^C]PHNO ([^11^C]-(+)-propyl-hexahydro-naphtho-oxazin) to be more sensitive to nicotine-induced DA release compared to [^11^C]raclopride [45]. To date, three studies have utilized [^11^C]PHNO PET in relation to nicotine administration and smoking-associated cues in humans. Specifically, following cigarette smoking, a 12% to 15% reduction in D2 and D3 receptor binding potential was observed compared to control conditions [46]. These findings are likely influenced by genetics, where, during abstinence, slow metabolizers of nicotine had lower [^11^C]PHNO-binding potential compared to fast metabolizers within the D2 regions of the striatum [47]. Interestingly, there was no change in [^11^C]PHNO-binding potential in the striatum of nicotine-dependent individuals following the presentation of tobacco-associated cues [48]. 

The effects of food cues and craving on neural activity and DA receptor binding have been widely investigated. fMRI studies demonstrated that food cues activate the amygdala, insula, orbitofrontal cortex, and striatum brain regions compared to neutral cues [49,50]. These cues of highly palatable foods activate similar reward neurocircuitry to tobacco use disorder [51]. Furthermore, food cravings are associated with increased bold-oxygen-level-dependent (BOLD) signals in the hippocampus, insula, and caudate [50], regions involved in craving, motivation, and memory [52]. PET studies demonstrated a positive association of food cravings and increased dorsal caudate and putamen regional cerebral blood flow [53], as well as an association of DA ligand binding within the dorsal striatum and feeding [54]. In summary, molecular imaging studies food cues provide supportive evidence of DAergic pathway activation. 

Similar to food addiction, fMRI studies have examined the neuronal activation patterns produced by nicotine. For example, dose- and time-dependent BOLD signal increases were observed within the anterior cingulate cortex, dorsolateral prefrontal cortex, and medial prefrontal cortex brain regions in cigarette smokers [55]. This pattern of brain activation is consistent with DAergic pathways innervating the frontal cortex, as well as evidence supporting acute nicotine’s role in positively enhancing reaction time, short-term memory, working memory, and attention [56]. Furthermore, smoking-cue fMRI studies demonstrated that nicotine-dependent smokers exhibited more BOLD signal activation than nonsmokers in the prefrontal cortex, ventral striatum, and NAcc brain regions [57,58]. In addition, contextual factors such as cigarette availability can affect neural activity, and variation in tobacco use disorder severity and genotype can modulate cue-induced activity [59].

#### 3.2.2. Neurobiology Unique to Tobacco Use Disorder

Nicotine is the main psychoactive component of tobacco, and it specifically acts as an agonist of nicotinic acetylcholine receptors (nAChRs) in the brain. nAChRs containing the α4 and β2 subunits are critical for mediating nicotine reinforcement, nicotine sensitivity, reward motivation, and DA release [60,61,62,63,64,65]. nAChRs are located throughout the brain, with highest density within the thalamus, basal ganglia, frontal cortex, cingulate cortex, occipital cortex, and insula [66,67]. Most importantly, nicotine stimulates the release of DA in the mesolimbic area, the corpus striatum, and the frontal cortex [68,69]. These DAergic pathways are critical in nicotine-induced rewarding behaviors [70], as well as in regulating reward, motivation, decision-making, learning, and memory [71].

Several preclinical and clinical studies of tobacco use disorder examined the effects of cigarette smoking and smoking-related behaviors on brain function, specifically β2-nAChR desensitization and subsequent upregulation [72]. Preclinical studies assessing nicotine administration in animals and postmortem human studies of smokers demonstrated β2-nAChR upregulation throughout the striatum, frontal cortex, anterior cingulate cortex, temporal cortex, occipital cortex, and cerebellum [73], suggesting greater levels of β2-nAChR desensitization and inactivation produced by long-term smoking or nicotine administration [74]. Brain imaging studies examining β2-nAChR availability in human smokers mimicked these preclinical finding [74,75,76,77]. Dysregulation of these brain regions following drug use is commonly associated with processing of drug cues and loss of inhibitory control, the primary contributing factor to relapse [78,79,80]. Furthermore, human postmortem studies of smokers with variable lifelong smoking histories and former smokers demonstrated that nAChR upregulation was reversible following abstinence [81]. Taken together, many preclinical and clinical brain imaging studies support the theory that long-term nicotine administration or chronic smoking can lead to nAChR desensitization and upregulation in smokers [82], but this upregulation is reversible following extended periods of smoking abstinence [81,83].

#### 3.2.3. Neurobiology Unique to Food Addiction

With the exception of the DAergic system, the literature on other neurocircuits implicated in food addiction is limited. To date, only a few fMRI studies have been conducted in individuals who met the YFAS cutoff threshold for food addiction. The first study by Gearhardt et al. (2011) found a positive correlation between food addiction scores and neural activation in the anterior cingulate cortex, medial orbitofrontal cortex, and amygdala when participants anticipated highly palatable foods [84]. Furthermore, upon tasteless food cue presentation, a negative correlation was observed between food addiction scores and activation in the caudate, a region implicated in reward motivation [84]. In a more recent fMRI study, Schulte et al. (2019) investigated food-cue effects on neural activity in obese women who either met the YFAS 2.0 threshold cutoff or did not [85]. When presented with highly palatable foods, participants with food addiction exhibited moderate, elevated activation in the superior frontal gyrus. Decreased activations were observed when minimally processed food cues were presented. Interestingly, participants in the control group had opposite responses in this region [85]. Most of the literature on the neurobiology of food addiction is derived from studies examining obesity; however, the findings from Schulte et al. (2019) presented food addiction as a unique phenotype within obesity.

Provided the limited neuroimaging research, it is theorized that dysregulation in the hypothalamus may also contribute to food addiction given its role as the main homeostatic regulation center for feeding behaviors. The hypothalamus integrates different hormonal and neuronal signals to control appetite and energy. This regulation system monitors body adiposity by using hormones such as leptin, insulin, and ghrelin [86]. Ghrelin, the “hunger peptide”, stimulates DAergic reward pathways, whereas leptin and insulin inhibit these circuits [49]. Several brain regions, such as the amygdala, hippocampus, insula, orbitofrontal cortex, and striatum, are also involved with the regulation of feeding and appetite [49]. These brain structures are involved in learning about food, allocating attention and effort towards food, conditioning reward with specific food cues in the environment, and integrating homeostatic information such as hunger with availability of food in the environment [49,71]. For a recent review of potential mechanisms for food addiction (in the presence of obesity) using a systems approach, see [87].

### 3.3. Role of the Gut Microbiome

#### 3.3.1. Parallels in the Role of Gut Microbiome in Both Tobacco Use Disorder and Food Addiction

As discussed in the section above, both food addiction and tobacco use disorder reflect an imbalance in the extended reward system in response to environmental stimuli. Peptides that regulate appetite such as glucagon-like peptide 1 (GLP-1), ghrelin, leptin, peptide YY, and neuromedin U are expressed throughout the brain reward circuitry, providing strong evidence that food addiction and tobacco use disorder share overlapping gut–brain axis mechanisms [88]. Endocrine signals play a significant role in reward regulation and dysregulation, which is a hallmark feature of all addictive disorders. The neuropeptides that have been studied most extensively are ghrelin and GLP-1.

There were only a few studies exploring the mechanism via which the gut microbiome affects the behavioral response to drugs of abuse [89,90,91]. Nonetheless, there is preliminary clinical and preclinical evidence of bacterial dysbiosis in response to drugs of abuse, which requires further investigation [92,93,94,95,96].

#### 3.3.2. Tobacco Use Disorder and the Gut Microbiome

The effect of smoking on the brain–gut axis and its behavioral implications has been largely unexplored. However, it has been shown that smoking induces specific changes in the microbiome. Furthermore, evidence suggests that smoking cessation induces an increase in microbial diversity [97,98], thereby reversing the negative effects of smoking and tobacco dependence on gut microbiota. A study by Biedermann et al. (2013) examined the association between smoking and gut microbiota in smokers without specific diseases [98] and showed that smoking cessation induced an increase in Firmicutes and Actinobacteria and a decrease in Bacteroidetes and Proteobacteria [98]. However, this study was conducted in only 10 subjects, and most of the participants developed an increased BMI following smoking cessation [98]. Previous studies showed that higher BMI is associated with increased Firmicutes and decreased Bacteroidetes in the gut compared to normal BMI [99,100]. Therefore, changes in gut microbiota following smoking cessation [98] might be associated with cessation-induced weight gain, as well as with smoking itself.

In a more recent large-scale cross-sectional study that included current, former, and never male smokers, smoking status influenced gut microbiota composition. Specifically, current smokers had a higher proportion of Bacteroidetes compared to never and former smokers, as well as lower proportions of Firmicutes and Proteobacteria compared with never smokers [89]. There were no observed differences in the composition of gut microbiota between never and former smokers, suggesting that smoking cessation allows gut microbiota composition to recover to pre-smoking status. The three groups did not differ significantly in terms of BMI or nutrient intake, thereby providing stronger evidence for the reversal of gut microbiota changes to normal upon smoking cessation.

Furthermore, a recent study compared the oral and gut microbiota in current smokers, current e-cigarette users, and healthy controls [101]. Tobacco smoking was associated with significant differences in the bacterial profiles in fecal, buccal, and saliva samples, while the e-cigarette users were no different to healthy controls. In keeping with previous studies, tobacco smokers had higher relative abundance of *Prevotella* and lower relative abundance of *Bacteroides* in their gut microbiota. This is in accordance with existing data demonstrating gut microbiotal changes following smoking cessation [98,102]

#### 3.3.3. Food Addiction and the Gut Microbiome

Eating behavior is regulated by both homeostatic and hedonic mechanisms in the central nervous system (CNS). These mechanisms involve orchestrated signaling from several sources including gut peptides, endocrine signals, and neuronal impulses, as well as signals from the gut microbiota. For example, ghrelin signals hunger and craving, putatively via amplification of DA signaling [103]. On the other hand, satiety is signaled by other intestinal hormones such as glucagon-like peptide 1 (GLP-1) and peptide YY [104]. Insulin also triggers hunger and increases the palatability of sugar. There is evidence that the gut microbiota can regulate insulin sensitivity through various mechanisms [105]. As discussed in the previous section, normal eating behavior is under the control of the extended reward network, which is involved in the processing of all rewarding stimuli including but not limited to food-related behaviors. These processes become maladaptive when the salience of a specific type of reward such as highly palatable food is greater than that of other stimuli and becomes preferred at the expense of other rewards, thereby leading to addiction-type behavior. At this point, the hedonic system becomes more prominent than the homeostatic system in the regulation of food intake. Therefore, eating behavior becomes driven predominantly by activation of the salience network of the brain, whereby food cues activate this network leading to increased attentional bias to the food cues at the expense of other cues. This in turn results in the uncontrolled overconsumption of highly palatable food.

While there is currently no evidence in humans that food addiction is caused by an altered gut microbiome or that it is driven by particular gut microbes or microbial metabolites, there is substantial evidence from rodent models that point to a role of the gut microbiome in food addiction-like behaviors. However, there is overwhelming evidence that a high-sugar, high-fat diet results in changes to the gut microbiome, which further “supports” addictive-like eating behaviors [87].

The few studies that examined the relationship between the gut microbiome and its metabolites with addictive-like eating behaviors have shown that tryptophan metabolites are implicated in modulating brain–gut–microbiome interactions [106]. In a recent study [107], the association between microbial profiles and tryptophan metabolites with food addiction was examined in a sample of human females with high BMI. The study found that there was a difference in the gut microbiome of females with food addiction versus those without, whereby levels of *Bacteroides* and *Akkermansia* were negatively associated with food addiction.

### 3.4. Psychological

#### 3.4.1. Childhood Adversity

Adverse childhood experiences (ACEs) have been shown to have deleterious effects on adult health [108,109,110]. To standardize the operationalization of childhood adversity within studies, Felitti et al. (1998) developed the ACE Survey, which quantifies an individual’s reports of exposure to abuse, neglect, and household dysfunction before the age of 18 [111]. These questions surveyed ACEs by asking about behaviors rather than subjective experiences of trauma. This survey originally encompassed seven categories including three of abuse and four of household dysfunction. More recent versions of the ACE study capture two additional categories of neglect and parental separation or divorce [112]. Cronholm et al. proposed the expansion of the concept of ACEs to include experiences such as witnessing violence, feeling discrimination, living in an unsafe neighborhood, experiencing bullying, and living in foster care to fully understand the influence of childhood adversity on adult substance use [113].

#### 3.4.2. Overlap in Childhood Adversity of Tobacco Use Disorder and Food Addiction

Childhood adversity influences various physiological and behavioral mechanisms that contribute to adult addictive behaviors. Studies demonstrate that chronic stress in childhood may cause changes in the nervous, endocrine, and immune systems. Alterations in these systems may lead to impairments in cognitive, social, and emotional development that predispose individuals with ACEs to adopt addictive behaviors [114,115,116]. The original ACE study reported that individuals who experienced four or more categories of adversity were 2.2 times more likely to be a current smoker and 1.6 times more likely to have a BMI ≥ 35 (severe obesity) [111]. This study did not evaluate food addiction. While the focus here is on tobacco use disorder and food addiction, individuals who report ACEs also engage in other addictive behaviors including alcohol abuse and drug use [111]. This suggests that childhood adversity may have significant and varying downstream effects on numerous adult health behaviors. While there is research on the relationships between childhood adversity and food addiction and between childhood adversity and tobacco use disorder, research is needed to better understand the overlap of food addiction and tobacco use disorder.

#### 3.4.3. Childhood Adversity and Tobacco Use Disorder

Using the ACE Survey, Felitti et al. (1998) produced a landmark paper describing the gradient relationships between the number of categories of childhood adversity and the prevalence of current smoking. Specifically, 6.8% of participants who reported no adversity (zero categories) and 16.5% of participants who reported four or more categories of adversity were current smokers. Participants who reported four or more categories of adversity were 2.2 times more likely to smoke than those who reported no adversity (odds ratio (OR) adjusted for age, gender, race, and educational attainment) [111]. Since then, a systematic review and a meta-analysis of 37 studies found that individuals who reported at least four categories of ACEs were more than twice as likely to be current smokers. Furthermore, there was a moderate association between childhood adversity and smoking [113].

#### 3.4.4. Childhood Adversity and Food Addiction

There is limited research into food addiction. One study identified childhood abuse as a risk factor for food addiction. This study of 57,321 adult women examined the association between child abuse (specifically, physical and sexual child abuse) and food addiction (defined as three or more clinically significant symptoms on the mYFAS). In this sample, over 8% of participants reported severe physical abuse in childhood, 5.3% reported severe sexual abuse, and 8% met the criteria for food addiction. Findings indicated that women with food addiction had a higher BMI than women without food addiction. Furthermore, severe physical and severe sexual abuse was associated with about 90% increased risk for food addiction (physical abuse: relative risk (RR) 1.92, 95% confidence interval (CI) 1.76 to 2.09; sexual abuse: RR 1.87, 95% CI 1.69 to 2.05). The RR for combined severe physical abuse and sexual abuse was 2.40 (95% CI 2.16 to 2.67) demonstrating the additive effects of adversity [117]. Childhood adversity has also been linked to disordered eating, including food addiction, obesity, and binge eating, which has overlapping characteristics with food addiction [111,118,119,120,121,122].

#### 3.4.5. Attachment Insecurity

Attachment theory describes how individuals internalize experiences with their caregivers to form mental representations of themselves and others. Individuals with high attachment anxiety tend to have negative self-views, are concerned about rejection, magnify their expressions of distress, and prefer close proximity to and support from a partner [123,124]. Individuals with high attachment avoidance tend to report positive self-views [123], suppress expressions of distress, and prefer emotional distance in relationships [124]. Individuals can be characterized by varying levels of attachment anxiety and avoidance, and both types of insecurity can co-occur [124]. The experience of childhood trauma is associated with both attachment anxiety and attachment avoidance in adulthood [125]. Attachment theory provides another framework to understand tobacco use disorder.

#### 3.4.6. Overlap in Attachment Insecurity of Tobacco Use Disorder and Food Addiction

Attachment insecurity may influence several processes that may in turn contribute to tobacco use disorder and food addiction. While there is research on the relationships between attachment insecurity and food addiction and between attachment insecurity and tobacco use disorder, research is needed to better understand the overlap of food addiction and tobacco use disorder. Attachment insecurity is related to affect regulation [126], i.e., “the process by which individuals influence which emotions they have, when they have them, and how they experience and express these emotions” [127]. Emotional regulation involves efforts to up- and downregulate positive and negative emotions [127]. High levels of attachment insecurity are associated with a deficit in affect regulation [128]. Individuals with high attachment insecurity may feel less capable of disengaging from negative feelings and in turn attempt to calm themselves through food or other substances. Food or other substances (e.g., tobacco, alcohol, or drugs), when consumed in order to reduce feelings of insecurity, have been called “external regulators of affect” [129]. Individuals with high attachment insecurity are more likely to use these “external regulators of affect” instead of utilizing more adaptive emotional regulation strategies. Attachment insecurity has been studied in the context of disordered eating, eating disorders, and obesity, but there is still limited research on food addiction [130,131,132].

#### 3.4.7. Attachment Insecurity and Tobacco Use Disorder

Insecure attachment patterns, specifically attachment anxiety, are associated with the use of tobacco and other drugs [133,134,135,136]. Attachment anxiety was associated with increased use of tobacco to reduce stress in college students [135]. In undergraduate and graduate students, significant differences in attachment patterns in tobacco users and nonusers were observed [122]. In a study of adults, findings suggested that attachment anxiety, but not attachment avoidance, was associated with current smoking [137]. In contrast, a study in adult women found that attachment avoidance was associated with being a current smoker [133]. It is currently unclear whether a particular dimension of attachment insecurity, such as attachment avoidance or attachment anxiety, is associated with tobacco use disorder.

#### 3.4.8. Attachment Insecurity and Food Addiction

Few studies examined the association of attachment insecurity and food addiction. One study of a national nonclinical sample of 1841 respondents in the Czech Republic found that attachment insecurity was associated with increased scores of mYFAS 2.0 [138]. In a study of 195 adult women from an eating disorder treatment center, the prevalence of food addiction was 83.6%, and the most frequently reported food addiction criteria were “clinically significant impairment or distress in relation to food”, “craving”, and “persistent desire or repeated unsuccessful attempts to cut down”. Within this sample, no differences in attachment insecurity were found between those meeting the criteria for food addiction and those who did not fulfill the criteria for food addiction [132]. As such, there may be a similarity between food addiction and eating disorders in terms of attachment patterns. The literature suggests that attachment insecurity is associated with unhealthy eating, including anorexia nervosa, bulimia nervosa, nonclinical levels of disordered eating, and obesity [130,131,139,140,141,142,143,144,145,146,147,148].

### 3.5. Treatment

#### 3.5.1. Treating Tobacco Use Disorder

There is a wide array of smoking cessation interventions that are well-established by evidence from several systematic reviews. Individualized treatments such as nicotine replacement therapy (NRT), varenicline, bupropion, behavioral supports, e-cigarettes, and combination therapies have positive outcomes on cessation rates and sustained abstinence. In addition, community- or government-level efforts such as prohibiting smoking in public spaces, advertising restrictions, and health warning labels contribute to reductions in smoking.

NRT is a common first-line, over-the-counter cessation aid that is available in forms such as the patch, lozenge, inhaler, and gum. NRT has a >6.5% sustained abstinence rate after 6 months, more than double that of placebo [149,150]. Compared to placebo, NRT shows no statistically significant differences in adverse events, except nausea, which has been listed as a common side effect [150]. Using the NRT patch together with another type of NRT (e.g., gum, lozenge, mist, or inhaler), can increase that rate by an additional 15–36% [151]. NRT dose and duration also affect quit rate. Quit success is positively correlated with higher-dose NRT patches (25 mg worn over 16 h or 21 mg worn over 24 h) compared to smaller-dose patches (15 mg over 16 h or 14 mg over 24 h) [151].

Varenicline is well established as achieving the highest quit and sustained abstinence rate of all cessation aids, with minimal risk for adverse effects (see, for example, [149,150,151]). Varenicline more than doubles the chances of quit compared to placebo [152,153], helping approximately 50% more people to quit and have sustained abstinence than the NRT patch, tablets, spray, lozenge, and inhaler, and 70% more than NRT gum [152]. The antidepressant bupropion has similarly high quit success, making it 52–71% more likely that a person will quit [154]. There have been concerns surrounding varenicline and bupropion’s linkage to psychiatric adverse events. A systematic review did not support this for varenicline, where the most frequently reported adverse event was nausea [153]. However, there is high-certainty evidence that unwanted mental health side-effects and adverse events linked to taking bupropion lead to lower medication adherence [154]. Furthermore, highest quit and sustained abstinence rates occur when pharmacotherapies are used in combination with behavioral supports. Behavioral therapy has been shown to increase effectiveness of pharmacotherapies by 83% to 97% across different care settings [155]. One systematic review compared the effects of brief physician advice to quit with offering assistance in the form of behavioral support or medication [156]. Physicians who offered assistance generated more quit attempts than those who gave advice to quit on medical grounds. Furthermore, when assistance was delivered in the form of motivational interviewing, a goal-oriented and patient-centered counseling approach that elicits motivation for change, abstinence rates were statistically significant and demonstrated up to 45% greater odds of smoking abstinence than control groups [157].

In recent years, e-cigarettes have become commonly used smoking cessation aids. While evidence is currently limited, the literature suggests the promise of e-cigarettes being an effective smoking cessation aid [158,159]. There is moderate-certainty evidence [using Grading of Recommendations Assessment, Development and Evaluation (GRADE)] from Cochrane systematic review that e-cigarettes with nicotine are more effective at helping people stop smoking for at least 6 months than NRT (three studies; 1498 participants), nicotine-free e-cigarettes (three studies; 802 participants), and no support or behavioral support alone (four studies; 2312 participants) [158]. Of the adverse events reported for e-cigarettes, throat and/or mouth irritation was the most commonly reported. Moderate-certainty evidence indicates the potential for results to change when more evidence becomes available. More research is needed to determine if e-cigarettes with nicotine are the preferred option for smoking cessation.

In summary, combination therapies that include either pharmacotherapy and behavioral support or a lower-risk nicotine product such as NRT or e-cigarettes with behavioral support increased quit attempts and boosted the level of sustained abstinence after one year. Organizational interventions also played a role in the sale and ease of promoting healthier smoking behaviors.

#### 3.5.2. Treatment Options for Food Addiction

There is currently no well-established treatment model for intervening on food addiction. This is unsurprising given that there is no formal recognition of food addiction as a neurological or behavioral disease within the DSM 5 due to the debate of perceiving food addiction as a (non)substance use disorder or as a behavioral addiction. Furthermore, the heterogeneity of addiction makes it difficult to translate addiction into a working treatment model.

In its contextualization along the spectrum of eating disorders within the scientific literature, there are notable behavioral treatment options. Psychosocial interventions for food addiction include reducing access to processed foods, reducing habit-based eating, removing restrictions on eating healthy foods, and behavioral therapies to improve emotional regulation and to help combat submission to cravings and emotional eating [160]. Furthermore, participation in an integrative and psychological weight management group demonstrated promise in treatment efficacy [161]. Learning about mindful eating, keeping a food diary and keeping track of body weight, creating and maintaining an exercise plan, and planning for social eating are tactics that are taught to enable the maintenance of healthy body weight [161]. A systematic review revealed that additional research is needed to develop and test the efficacy of these types of interventions within the context of food addiction [162].

Noninvasive brain stimulation has been used most frequently for the treatment of addictions such as tobacco use disorder [163]. Transcranial direct current stimulation (tDCS) in particular, is a safe, economical, and accessible means of modifying neural activity [163]. A systematic review highlighted that tDCS significantly improved the symptoms of food addiction by reducing food cravings brought on by visual stimuli [164]. Craving was measured before and after stimulation using visual analogue scales, eye tracking, or the Food Craving Questionnaire—State, and tDCS was found to significantly repress the desire to eat, leading to less food consumption [164]. However, due to underpowered studies and the complexity of addiction, more research is necessary to make any definitive comments about the success of tDCS on food addiction. Tailoring neuromodulating interventions to individuals or subgroups, on the basis of cognitive and neural profiling, might prove to be useful [163]. Since food addiction often presents with comorbidities, current research suggests using evidence-based interventions to address other conditions first [162].

## 4. Current and Future Research Directions

Evidence demonstrates that individuals can expect to gain an average of 4 to 5 kg of weight after successfully achieving smoking cessation [165,166]. For many tobacco users, this potential increase in weight can be a substantial obstacle when attempting smoking cessation [167,168], thereby leading to continued tobacco use. One possible explanation for post-smoking cessation weight gain is that quitting smoking increases the desire to consume highly palatable, high-calorie foods. Consequently, the authors of this review developed a feasibility study with the purpose of examining the association between food addiction symptoms and smoking behavior among individuals seeking treatment for tobacco use disorder. This feasibility study recruited individuals from the Nicotine Dependence Clinic at the Centre for Addiction and Mental Health (CAMH) who had yet to begin their smoking cessation treatment. Those individuals who provided consent to participate in the study were asked to complete a survey which included the FTND and mYFAS questionnaires.

The sample of this feasibility study included 51 participants seeking treatment for tobacco use disorder. The majority of participants in this convenience sample were male (58.8%). Two-thirds of the sample consumed <20 cigarettes per day (CPD). The prevalence of individuals meeting the cutoff for food addiction was 11.8%. The average symptom count for food addiction was 1.5 ± 1.8 (standard deviation (SD)) symptoms.

Spearman rank correlation coefficients were conducted to examine the association between tobacco use disorder, specifically CPD, and food addiction symptom count, as measured by the mYFAS. No significant association was observed between CPD and food addiction symptom count for the overall sample (*r*_s_ = 0.21, *p* = 0.14) or by sex (male: *r*_s_ = 0.20, *p* = 0.28; female: *r*_s_ = 0.24, *p* = 0.31).

The results of this feasibility study, even though underpowered, represent an important initial step in developing future research. Using a cross-sectional study design, we were unable to observe a significant association between food addiction symptom count and CPD. The findings from another cross-sectional study examining the association between smoking and food addiction found that male smokers report twice as many YFAS symptoms compared to male nonsmokers [169]. However, among females, no relationship was observed between food addiction symptoms and smoking [169]. While this study provides some evidence of an association between smoking and food addiction, more research is needed. Applying a longitudinal study design and examining how changes in smoking behavior relate to changes food addiction symptoms could provide greater insight into the relationship between smoking cessation and weight gain. Furthermore, the feasibility study also revealed that the prevalence of food addiction may be lower among individuals seeking smoking cessation treatment compared to the general population, suggesting, perhaps, that food addiction may have a minor role in post-cessation weight gain. Therefore, examining other concepts such as the role that the gut microbiome may have on smoking cessation weight gain could be promising. However, it is also plausible that achieving smoking cessation could result in the adoption of an addictive behavior involving the overconsumption of highly palatable foods, a process known as addiction transfer [170].

E-cigarettes have been demonstrated to be effective smoking cessation aids [158]. As outlined above, e-cigarette users have gut and oral microbiomes that are more similar to healthy controls than to tobacco smokers [103]. Given that *Bacteriodes*, the bacterium that is most commonly significantly decreased in smokers compared to healthy controls and e-cigarette users, is also implicated in obesity [171], there is the potential that switching smokers to e-cigarettes may decrease their risk of cessation-related weight gain. This hypothesis has not yet been tested, and there is no clear evidence that smokers who switch to e-cigarettes avoid weight gain. As such, these are important research gaps to address.

## 5. Limitations

This was a narrative review; thus, limitations with respect to the scope of literature covered are present. The authors made every attempt to be systematic in their initial literature search, but they may have missed some important publications. Narrative reviews are also subject to bias, but the authors did their best to mitigate this by using systematic approaches to their literature search.

## 6. Conclusions

Food addiction and tobacco use disorder share similar but not identical neurological, physiological, and behavioral abnormalities. We attempted to summarize these similarities between the two disorders where there is evidence of their existence. Differences between the two disorders should not lead one to conclude that food addiction is not a “real” disorder. As argued in a recent perspective [172], core features of addiction can differ dramatically depending on which substance is being used, reflecting different underlying neurobiological processes at play. For example, the pattern of consumption and symptoms of withdrawal exhibited in cocaine use disorder completely different to what is seen in tobacco use disorder.

Addiction is a complex disorder that we are only beginning to understand at a system level. The role of the gut–brain axis in brain reward mechanisms related to addiction needs to be further researched. In addition, more attention needs to be paid to the role that early life events have on the risk of developing an addictive disorder. Taking learnings from different types of addictive behaviors such as food addiction, as well as newer “behavioral addictions” such as internet gaming, will help to further our understanding of the underlying mechanisms common to all addictive disorders. This will in turn pave the road toward effective treatment options. Exploring the commonalities between tobacco use disorder and food addiction is a step in this direction.
